# Using Health-Related Quality of Life to Identify the Incident Cardiovascular Disease Risk

**DOI:** 10.1093/geroni/igab046.2335

**Published:** 2021-12-17

**Authors:** Aung Zaw Zaw Phyo, Joanne Ryan, David A Gonzalez-Chica, Nigel P Stocks, Christopher M Reid, Andrew M Tonkin, Robyn Woods, Rosanne Freak-Poli

**Affiliations:** 1 Monash University, Melbourne, Australia, Melbourne, Victoria, Australia; 2 Monash University, Melbourne, Victoria, Australia; 3 University of Adelaide, Adelaide, South Australia, Australia

## Abstract

Previous studies have revealed that poor health-related quality of life (HRQoL) is associated with a higher risk of hospital readmission and mortality in patients with cardiovascular disease (CVD). The association between HRQoL and incident CVD is still limited for general older people. This study explored the associations between baseline HRQoL and incident and fatal CVD in community-dwelling Australian and the United States older people enrolled in ASPREE clinical trial. A cohort of 19,106 individuals aged 65 to 98 years, who were initially free of CVD, dementia, or disability, were followed between March 2010 and June 2017. The SF-12 questionnaire was used to assess HRQoL, and the physical (PCS) and mental component scores (MCS) of SF-12 were derived using norm-based methods. Incident major adverse CVD events included fatal CVD (death due to atherothrombotic CVD), hospitalizations for heart failure, myocardial infarction or stroke. Analyses were performed using Cox proportional-hazard regression. Over a median 4.7 follow-up years, there were 922 incident CVD events, 203 fatal CVD events, 171 hospitalizations for heart failure, 355 fatal or nonfatal myocardial infarction and 403 fatal or nonfatal strokes. A 10-unit higher PCS, but not MCS, was associated with a lower risk of incident CVD (HR=0.86, 95%CI 0.79-0.92), hospitalization for heart failure (HR=0.72, 95%CI 0.60-0.85), and myocardial infarction (HR=0.85, 95%CI 0.75-0.96). Neither PCS nor MCS was associated with fatal CVD events or stroke. Physical HRQoL can be used in combination with clinical data to identify the incident CVD risk among community-dwelling older people.

